# Impact of the COVID-19 Pandemic on Lung Cancer Screening and Diagnosis: A Systematic Review

**DOI:** 10.3390/cancers18142238

**Published:** 2026-07-13

**Authors:** Anastasia Savva, Panayiota Christodoulou, Charalambos Michaeloudes, Paraskevi A. Farazi

**Affiliations:** School of Medicine, European University Cyprus, 2404 Nicosia, Cyprus; anastasiasavva99@gmail.com (A.S.); pa.christodoulou@euc.ac.cy (P.C.); c.michaeloudes@euc.ac.cy (C.M.)

**Keywords:** COVID-19 pandemic, coronavirus, lung cancer, diagnosis, screening, impact

## Abstract

**Background/Objectives**: The global health crisis of the COVID-19 pandemic in 2020 severely impacted healthcare systems, particularly affecting cancer patients, including those with lung cancer. Understanding the pandemic’s effects on lung cancer diagnosis is important for providing guidelines for future similar crises. **Methods**: A comprehensive search was conducted using the SCOPUS and PubMed databases, including keywords such as ‘COVID-19 pandemic,’ ‘lung cancer,’ ‘diagnosis,’ and ‘screening.’ In the initial screening phase, studies were selected based on their title and abstract, followed by the removal of duplicates. A second round of full-text screening was then performed using predefined inclusion and exclusion criteria. Specific checklists were applied to ensure methodological quality. **Results**: Out of 564 articles, 78 met the inclusion criteria and were grouped according to changes in lung cancer incidence, screening and diagnostic procedures, and staging during the COVID-19 pandemic compared to pre-pandemic. Multiple studies reported an average decline of 20% in newly diagnosed lung cancer cases, alongside significant decreases in screenings, follow-ups, and diagnostic procedures. In contrast, only two studies reported an increase in lung cancer incidence, and three studies reported increases in screening or diagnostic activity. Eight studies reported a shift toward more advanced-stage diagnoses and fewer early-stage detections, while four studies observed a decrease in advanced-stage lung cancer during the pandemic compared to pre-pandemic years. **Conclusions**: Studies show that overall, the COVID-19 pandemic disrupted lung cancer diagnosis in many countries, highlighting the need for stronger healthcare systems that can support crisis response and equitable care.

## Simple Summary

The COVID-19 pandemic brought significant challenges to both individuals and healthcare systems worldwide. This systematic review aimed to evaluate how the pandemic disrupted lung cancer diagnosis. Among the 78 included studies, most reported an average decline of approximately 20% in newly diagnosed cases, along with significant reductions in screening, follow-up, and diagnostic procedures, although a few studies showed opposite trends. This review summarizes global evidence and highlights the importance of maintaining essential healthcare services during crises.

## 1. Introduction

The outbreak of SARS-CoV-2, leading to the COVID-19 pandemic, posed severe challenges to global healthcare systems, significantly impacting the management of diseases, including cancer. Governments enforced measures like travel restrictions, lockdowns, mask mandates, and workplace safety protocols, including vaccination [1]. More than 100 countries imposed full or partial lockdowns, affecting billions of people. Measures eased during mid-2020 but were reinstated with successive virus surges. This cycle of tightening and easing restrictions persisted until May 2023, when the WHO lifted its public health emergency of international concern (PHEIC) status. These restrictions have affected cancer screening and diagnosis, including lung cancer [2,3].

Lung cancer is the most prevalent cancer in men and the second most prevalent in women [4]. In the U.S., it ranks as the second leading cause of death after heart disease, with nearly 200,000 new cases and 126,000 deaths reported in 2020 [5]. Early detection significantly affects survival rates, as only about 10% of patients survive beyond 10 years without early diagnosis [6]. Lung cancer stems from genetic mutations in lung cells, often involving the EGFR, KRAS, or ALK genes, leading to abnormal cell growth and tumor formation [4]. Smoking is the primary risk factor, though non-smokers can also be affected by factors like air pollution, radiation exposure, or a family history of lung cancer [4]. Lung cancer comprises two main types: small cell lung cancer (SCLC), and non-small cell lung cancer (NSCLC), which is more common and includes subtypes like adenocarcinoma and squamous cell carcinoma [4].

Most cases are diagnosed at advanced stages due to early asymptomatic development. Diagnosing lung cancer involves a combination of physical exams and imaging, such as X-Rays, Positron Emission Tomography (PET), Computed Tomography (CT) scans, and biopsies [4]. High-risk individuals, namely adults between 50 and 80 years old, with a smoking history of ≥20 pack-years, who are current smokers or have quit within the last 15 years, benefit from annual low-dose CT (LDCT) screenings, as recommended by the U.S. Preventive Services Task Force (USPSTF). The USPSTF recommendations informed the American Cancer Society 2023 guideline update, adopting the same eligibility criteria but removing the 15-year smoking cessation requirement, hence expanding screening eligibility [5,7]. Early screening helps identify cancer at stages I or II, when treatment is most effective. Despite the benefits, screening programs remain underdeveloped in many regions and are inaccessible to certain populations [8,9]. Staging is crucial for managing and determining treatment for lung cancer. The tumor-node-metastasis (TNM) lung cancer classification system is based on the size and local extent of the primary tumor (T), regional lymph node involvement (N), and the presence or absence of distant metastasis (M) and ranges from stage I (localized disease) to stage IV (advanced metastatic disease) with sub-categories of these as defined by the 9th TNM edition staging system that introduced significant changes in the T component, different categorizations of the N component and subdivision of N2, and M components [10,11,12].

Notable shifts in care and lung cancer diagnosis were reported during the COVID-19 pandemic. Specifically, a publication by the American Society of Clinical Oncology (ASCO) reported a notable decline in the annual incidence of new lung cancer diagnoses during the pandemic, compared to the pre-pandemic period, likely due to reduced regular screening [13,14]. Robust screening programs can support consistent lung cancer screening during major health crises, such as the COVID-19 pandemic, helping health systems reduce diagnostic delays. A robust screening program is not defined simply by increased volume of screening, but by implementing more targeted and effective screening. For example, during the COVID-19 pandemic, expert panels issued guidance recommending prioritizing individuals who are at high risk and for whom a delay in screening would be detrimental [15]. Therefore, when face-to-face medical care is disrupted during pandemics, strategies that risk-stratify and identify high-risk individuals who should be prioritized for screening should be implemented. This ensures more efficient allocation of resources and prevents delays for patients who require urgent screening. Therefore, understanding the factors contributing to efficient and inclusive screening programs would be beneficial for improving public health responses to these crises.

Given the disruptions that arose from the COVID-19 pandemic and reports noting changes in lung cancer incidence rates during the pandemic, this review aims to assess the effects of the COVID-19 pandemic on lung cancer diagnosis. More specifically, the review includes studies focusing on the impact of the pandemic on lung cancer incidence, screening, and diagnostic procedures, as well as the stage of cancer. Furthermore, the review explores the time trends and the effects of patient characteristics on the impact of the pandemic. By examining these factors, this review hopes to contribute insights that would motivate the development of predictive models to identify high-risk individuals that should be prioritized for screening, the development of non-invasive lung cancer screening tests, and building more robust and patient-focused oncology care systems capable of enduring future health crises.

## 2. Materials and Methods

### 2.1. Study Information

This systematic review was conducted following the Systematic Review Checklist (PRISMA) and was registered in PROSPERO (ID: CRD42024519501).

### 2.2. Eligibility Criteria

The inclusion criteria for the studies were: peer-reviewed, any type of lung cancer diagnosis at any stage, assessment of the impact of the COVID-19 pandemic specifically on the diagnosis of the disease (i.e., comparing either pre- and during the pandemic era or during and post the pandemic era), and written in the English language. The study designs that were eligible for inclusion were observational (cohort studies, case–control studies, cross-sectional studies) or interventional (clinical trials). The exclusion criteria were as follows: any article irrelevant to the topic and the keywords of the study, published in a different time frame other than the COVID-19 pandemic, not reporting on diagnosis, focusing on any other type of cancer, and not in the English language.

### 2.3. Information Sources

The Scopus and PubMed databases were searched from February 2024 up to November 2025.

### 2.4. Search Strategy

A systematic approach was employed to identify relevant articles from PubMed and SCOPUS, focusing on the impact of the COVID-19 pandemic on lung cancer diagnosis. The search included the following keywords “COVID-19 pandemic,” “lung cancer”, “diagnosis”, “impact”, “screening”, used with the AND Boolean operator. To capture a comprehensive dataset, synonyms such as “coronavirus”, “NSCLC”, “SCLC”, “early detection”, “effect”, and “consequences” were also used with the OR Boolean operator. The exact search strategy used was: (“COVID-19 pandemic” OR coronavirus) AND (“lung cancer” OR NSCLC OR SCLC) AND (screening OR “early detection” OR diagnosis) AND (consequences OR impact OR effect).

The literature search of PubMed and Scopus was conducted initially in February 2024 and updated in November 2025, using the same databases, keywords, and inclusion/exclusion criteria.

### 2.5. Selection Process

**Figure 1:**
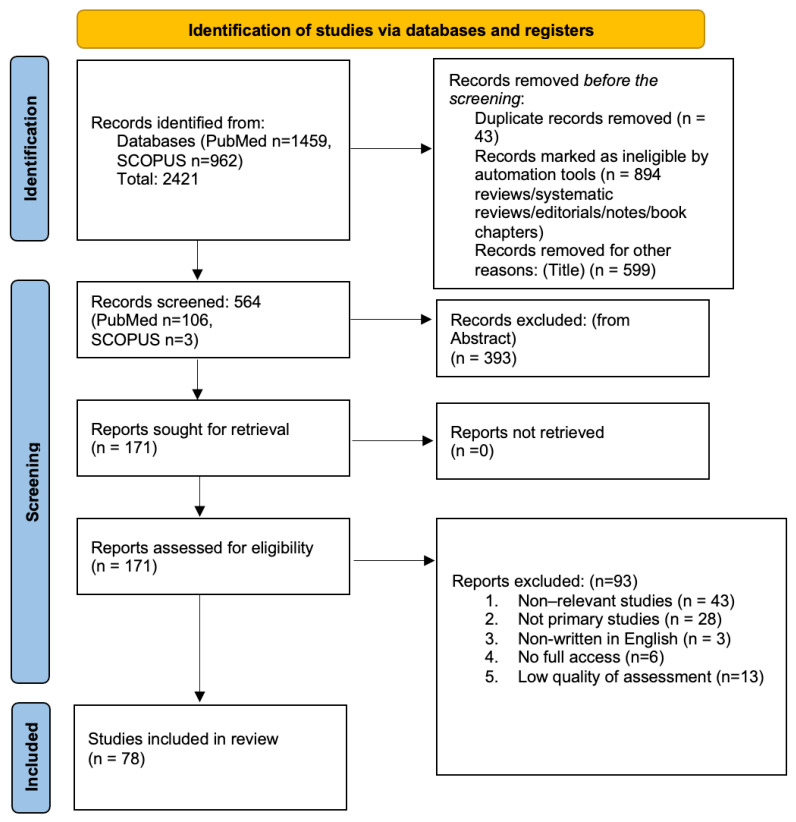
PRISMA 2020 flow diagram illustrating the flow of the screening process.

Searches were performed up to November 2025, yielding 2421 articles. Automation tools excluded 894 articles such as reviews, editorials, or book chapters, and 599 more were omitted for having irrelevant titles. After removing 43 duplicates, 564 articles were screened further. Independent reviewers screened all titles and abstracts per eligibility criteria, with four reviewers working in pairs (PC and CM, PF and AS). Initially, 393 abstracts were excluded as not meeting the study’s scope, with 171 articles remaining for full-text evaluation. Ultimately, 78 articles were identified as meeting the predefined inclusion/exclusion criteria, following a consensus between reviewers (Figure 1). Where there was disagreement between the two reviewers, a third reviewer would review the article independently, and all reviewers would discuss and come to a consensus.

### 2.6. Data Collection Process

Data extraction was carried out by two independent reviewers and included key information like author, publication year, country, sample size, demographics, and main findings related to the studied outcomes that would enable assessment of the impact of the COVID-19 pandemic on lung cancer diagnosis. The main findings were divided into the following groups: impact of the COVID-19 pandemic on lung cancer incidence, impact of the COVID-19 pandemic on lung cancer screening and lung cancer diagnostic procedures, and impact of the COVID-19 pandemic on the stage of lung cancer diagnosis. Additional results regarding the timeline of the impact of the pandemic as well as patients’ characteristics, were reported. All the studies compared their results with the year/years before the COVID-19 pandemic or year/years after the COVID-19 pandemic.

### 2.7. Data Items

The PICOS strategy used was based on the following designations: P = all patients diagnosed with any stage of lung cancer, I = COVID-19 pandemic, C = pre-pandemic years or post-pandemic years, O = (1) lung cancer incidence, (2) lung cancer screening (LCS), (3) lung cancer diagnostic procedures, and (4) stage of lung cancer diagnosis, S = primary studies only (observational such as case–control, cohort studies and interventional, such as RCTs). Thus, information regarding the comparison of pre- vs. during or pre- vs. after the pandemic was collected for the above outcomes.

### 2.8. Study Risk of Bias Assessment

The risk of bias assessment was conducted by two independent researchers using the STROBE or CONSORT checklists, accordingly, ensuring articles met quality standards. Disagreements were resolved through discussion. Thirteen articles were excluded for lacking statistical analysis or failing checklist criteria [16].

### 2.9. Effect Measures

The effect measures used were percentage change in the outcomes pre- and during the pandemic period.

### 2.10. Synthesis Methods

Studies assessing the same outcome were grouped and graphed together to allow for inter-study findings and identify trends in the percentage change in the outcomes between the two periods. The comparisons were between studies and countries to identify any regional differences in the impact of the COVID-19 pandemic on lung cancer incidence and diagnosis.

## 3. Results

### 3.1. Selection of Studies and Their Characteristics

After the first round of screening, we had 171 articles left for full-text assessment. Of these, 93 were excluded for various reasons: 43 for not aligning with the study objectives, 28 for not being primary studies, 13 for low quality, 6 for lack of full-text access, and 3 for being in a language other than English. Ultimately, 78 articles were included in the review: 39 retrospective cohort studies, 16 cross-sectional studies, 2 case–control studies, 11 descriptive studies, 6 population/registry-based studies, 3 single-center analyses, and 1 time-series analysis (Figure 1). The eligible articles that were considered for analysis were divided into the following groups based on the following outcomes: (1) impact of the COVID-19 pandemic on lung cancer incidence, (2) impact of the COVID-19 pandemic on lung cancer screening or lung cancer diagnostic procedures, and (3) impact of the COVID-19 pandemic on the stage of lung cancer diagnosis. The timeline of the pandemic’s impact, along with the characteristics of the patients, was also reported.

### 3.2. The Impact of the COVID-19 Pandemic on Lung Cancer Incidence

**Figure 2:**
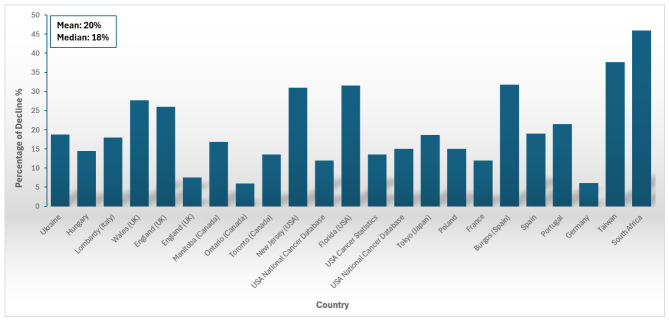
Percentage of decline in lung cancer incidence during the COVID-19 pandemic (2020) compared to pre-pandemic years in various countries/areas [17,18,19,20,21,22,23,24,25,26,27,28,29,30,31,32,33,34,35,36,37,38,39].

Multiple studies from diverse geographic regions, including Ukraine, Hungary, Italy, the United Kingdom, Canada, the United States, Japan, Poland, France, Spain, Portugal, Germany, Taiwan, and South Africa, consistently reported a decline in lung cancer incidence or in the number of newly diagnosed lung cancer cases during the COVID-19 pandemic compared with pre-pandemic years (Figure 2) [17,18,19,20,21,22,23,24,25,26,27,28,29,30,31,32,33,34,35,36,37,38,39].

Across these studies, the average reduction in lung cancer incidence was approximately 20%, reflecting a substantial global impact. Importantly, these countries collectively represent a large proportion of the world’s population and health care systems, suggesting that the observed decline in lung cancer diagnoses during the pandemic affected a significant share of the global population rather than being confined to isolated regions. Additionally, the median decline in lung cancer incidence was 18.0%, indicating that the overall estimate was not substantially influenced by extreme values.

However, one of these studies, by Mangone et al., in Northern Italy, showed specifically that the decrease was observed only in men [40]. Conversely, in a study in France by Demoustier et al., lung cancer incidence showed a 14% reduction among women only, a trend that predated lockdown measures, with no corresponding decline in men [33]. These studies were not included in Figure 2 as they reported changes in lung cancer incidence restricted to sex-specific subgroups (men or women only) rather than overall population estimates. Additionally, Ramanakuma et al. (Quebec, Canada), Mora et al. (Catalonia), Vardhanabhuti et al. (Hong Kong), Cantini et al. (Milan), and Barclay et al., (UK) showed that there was a reduction in new lung cancer cases during the COVID-19 pandemic, albeit it was not statistically significant [41,42,43,44,45]. Another study in Turkey showed that the number of patients diagnosed with lung cancer during the pandemic period was lower compared to the pre-pandemic period [46].

In contrast, two studies showed an increase in the number of lung cancer cases during the pandemic compared to the years before. More specifically, one study in Japan showed an increase in the number of SCLC cases during the pandemic year (*p* = 0.035) but no changes in the number of NSCLC cases between 2018 and 2021 [47]. Another study in Florida (USA) showed a slight, but statistically significant, increase in lung/bronchus cancer diagnoses, from 228,150 in 2019 to 228,820 in 2020 (0.29% increase) and 235,760 in 2021 (3.34% increase). The incidence rate decreased slightly in 2020 (69.15 per 100,000 person-years) compared to 2019 (69.51 per 100,000) but increased to 70.91 per 100,000 in 2021, a statistically significant rise (*p* < 0.001) compared to 2019. They suggested that the reduction in cancer screenings during the pandemic likely delayed cancer detection, allowing pre-cancerous conditions to progress, thus increasing cancer diagnoses [48]. Meanwhile, the study in Northern Italy that observed a decrease in lung cancer incidence among men (−9.6%), conversely reported an increase among women (+3.6%) [40]. Mitchell et al. in New Zealand and Kathleen et al., in the Northern region of Manitoba showed a non-significant increase in new lung cancer diagnoses during the pandemic [23,49].

A study conducted in Germany by Tarawneh et al. showed that the lung cancer incidence remained stable during the pandemic compared to pre-pandemic years [37]. Similarly, Park et al., in Korea, showed that despite the lower number of outpatient visits, the rate of new lung cancer diagnoses did not decrease significantly [50].

### 3.3. The Impact of the COVID-19 Pandemic on Lung Cancer Screening and Lung Cancer Diagnostic Procedures

**Figure 3:**
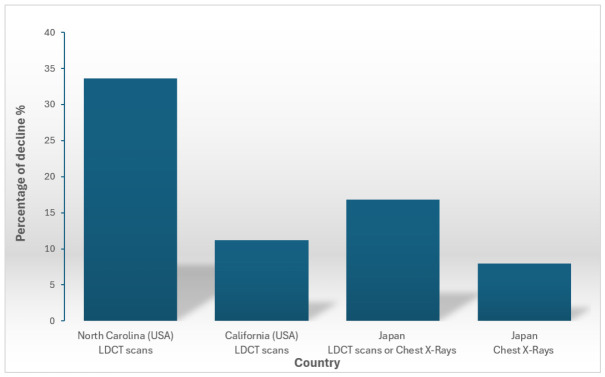
Percentage of decline in LDCT scans for lung cancer screening during the COVID-19 pandemic compared to pre-pandemic years in North Carolina (USA), California (USA), and Japan [51,52,53,54,56]. Additional studies from Ohio (USA) [53] and Yale New Haven Health Network [55] are not included in the figure because the studies’ data were not directly comparable to the others.

Several studies reported significant declines in lung cancer screening and diagnostic procedures during the COVID-19 pandemic compared with pre-pandemic years. In the United States, including data from California, North Carolina, and Ohio, reductions in lung cancer screening using low-dose computed tomography (LDCT) were consistently observed during the pandemic period [51,52,53]. Notably, in the United States, the largest reduction in lung cancer screening using low-dose computed tomography (LDCT) was reported in North Carolina [52], with a 33.6% decline, whereas a more modest decrease was observed in California (11.2%) during the pandemic, compared with the pre-pandemic period (Figure 3) [51].

Furthermore, in Japan, participation in lung cancer screening (which does not define whether screening was by CT scans or chest X-Rays) declined substantially during the COVID-19 pandemic. The number of individuals undergoing screening decreased from 7.92 million in 2019 to 6.59 million in 2020, representing a 16.8% reduction, likely attributable to pandemic-related disruptions (Figure 3) [54].

Consistent with this finding, another USA-based study from Yale healthcare network reported a sharp decline in CT-based lung cancer screening during the early phase of the pandemic, with screening volumes falling by approximately 70% between April and June 2020 compared with pre-pandemic levels [55]. However, screening activity rebounded rapidly, surpassing baseline levels by mid-2020 and demonstrating sustained growth thereafter (+4.5% in 2020, +69.6% in 2021, and +27.0% in 2022), with no subsequent declines observed during later COVID-19 surges [55]. In contrast, a study in Japan revealed that participation in chest radiography (chest X-Ray) for lung cancer screening showed a more modest but persistent decline, decreasing by approximately 8% in 2021 compared with the pre-pandemic period (Figure 3) [56].

**Figure 4:**
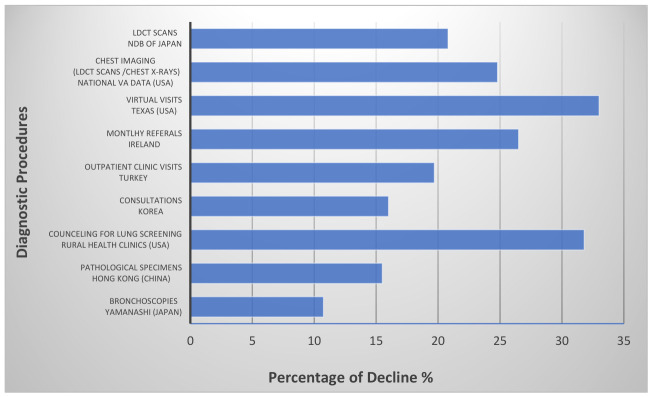
Percentage decline in lung cancer diagnostic procedures during the COVID-19 pandemic compared to pre-pandemic years [46,50,57,58,59,60,61,62,63].

Regarding lung cancer diagnostic procedures and hospital visits, several studies from Turkey, Korea, Japan (NDB of Japan, Yamanashi), China (Hong Kong), the National Veterans Affair (VA) data (USA), Ireland, and Texas (USA) have reported a decrease in lung cancer diagnostic procedures and follow-up among both outpatients and inpatients, including bronchoscopy, LDCT scans (but not for screening), pathological specimen collection, chest imaging (LDCT scans or chest X-Rays), specialist consultations, and clinical visits (Figure 4) [46,50,57,58,59,60,61,62,63].

An additional study conducted in Ireland by Ghassemi-Rad et al., reported that compared to pre-pandemic period, average monthly referrals and RALC (Rapid Access Lung Cancer Clinic) reviews decreased by 26.5% and 9.5%, respectively, in period II (March 2020 to February 2021); however, timeliness of care improved, with significantly shorter intervals from referral to CT (~22% reduction) and to surgery (~27% reduction), and no delays in referral-to-review or diagnosis [62].

Furthermore, another study in Texas by Gwin et al., which reported the virtual visits during the early COVID-19 period (March–August 2020), showed that nearly half of all low-dose CT (LDCT) orders (48%) were placed. In contrast, after this period, only 15% of LDCT orders were placed virtually, representing a 33% reduction in virtual ordering. Despite this shift back toward in-person care, LDCT completion rates remained consistently high (95–97%) with no significant differences observed by visit type [63].

Conversely, three studies reported an increase in lung cancer screening, and three studies reported an increase in lung cancer diagnostic tests during the pandemic compared to pre-pandemic. In New Jersey (USA), reports showed a significant increase in CT scan screening procedures from July to December 2020 compared to pre-pandemic [26]. Another study by Englum et al., of Veterans’ electronic medical records in the US, showed CT chest screening rates during the pandemic that exceeded those in the pre-COVID era [61]. A study, at the national level in the United States, showed that lung cancer screening utilization increased from 16.3% in 2019 to 19.4% in 2020 (approximately 19% relative increase) and remained above pre-pandemic levels in 2021 (+12%) and 2022 (+11%), despite a modest decline following the initial pandemic peak [64].

When it comes to diagnostic procedures, in Poland, a study showed that high-resolution CT (HRCT) scans in the emergency department (ED) increased during the pandemic compared to the pre-pandemic year 2019. This finding showed that the pandemic led to more frequent use of HRCT scans, which, despite the challenges, had the incidental effect of leading to earlier oncological diagnoses [65]. Along the same lines, a study in Leicester (UK) reported a higher percentage of emergency department presentations due to other health problems that eventually led to lung cancer referrals during the pandemic [66]. In Northern Italy, while histological confirmation for lung cancer was decreased during the pandemic, a significant increase in cytology use for lung cancer diagnoses was observed (Supplementary Material Figure S1) [40].

Some studies did not report statistically significant differences in lung cancer screening and lung cancer diagnostic procedures, even though the majority showed a declining trend. More specifically, six studies reported steady rates of lung cancer screening during the pandemic compared to pre-pandemic years. Fedewa et al. (USA) found that national screening rates were maintained, with slight decreases in some states [67]. Machii et al. (Japan) observed a non-significant decrease [68], while Magarinos et al. (USA) found no significant difference in in-person screenings despite the use of telemedicine during the pandemic [69]. Lou et al. (USA) [26], and Joung et al. (Chicago) reported no significant changes in CT scans or screening volumes [70]. In Georgia, overall lung cancer screening attendance remained stable during the COVID-19 years (79.4%) compared with the pre-COVID period (80.1%), representing a non-significant ~0.9% relative decrease (*p* = 0.92). However, a marked short-term disruption was observed in April 2020, with a 19.4% decline [71]. Regarding lung cancer diagnostic procedures, one study in Lombardy (Italy) reported that, despite the COVID-19 pandemic, the number of patients undergoing transthoracic CT-guided FNA biopsy remained unchanged [72].

### 3.4. The Impact of the COVID-19 Pandemic on the Stage of Lung Cancer Diagnosis

**Figure 5:**
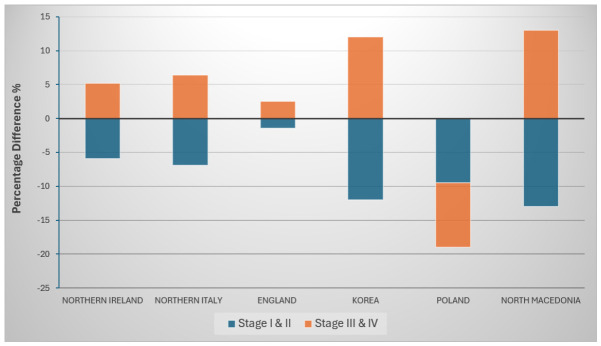
Percentage difference in lung cancer staging during the COVID-19 pandemic in different areas compared to pre-pandemic. The figure shows an overall increase in lung cancer diagnosis at late stages and a decrease in lung cancer diagnosis at early stages during the pandemic compared to pre-pandemic years [21,40,50,73,75,76]. Additional studies from Italy [44] and Germany [74] are not included in the figure because the studies’ data were not directly comparable to the others.

Eight studies showed both an increase in advanced-stage lung cancer diagnoses and a decrease in early-stage lung cancer diagnoses during the pandemic compared to pre-pandemic. The studies included areas in the UK, Italy, Korea, Northern Ireland, Germany, Poland, and Macedonia (Figure 5) [21,40,44,50,73,74,75,76]. Four articles, including areas in Milan (Italy), Japan, Canada, and Michigan (USA), reported only an increase in the advanced stages of lung cancer during the pandemic (Supplementary Material Figure S2) [44,47,77,78]. One article from Leicester (UK) reported a decrease in the early stages of lung cancer during the pandemic [66].

In contrast, three studies from areas including Turkey, Denmark, and Brazil showed that the diagnosis of advanced stages of lung cancer was decreased during the COVID-19 pandemic compared to pre-pandemic [46,79,80]. An additional study from Japan reported mainly reductions in the advanced stages, with a significant decrease in stage IV later in 2020 [31].

On the other hand, seven studies reported non-significant changes in the stage of lung cancer at diagnosis. More specifically, Tran et al. (Ontario, Canada) [81], and Keogh et al. (Ontario, Canada) found no significant changes in cancer stages or tumor characteristics at diagnosis during the pandemic compared to pre-pandemic [82]. Kizilirmak et al. (Turkey) and Krzyzaniak et al. (Poland) also reported no significant stage differences at diagnosis [46,65]. Martinez et al. (Spain) noted earlier-stage diagnoses, but the differences were not statistically significant [83]. Berrian et al. (Florida, USA) observed a slight increase in metastatic lung cancer, but the difference was not significant for all cases [28]. Ramanakumar et al., in Quebec (Canada), found that advanced stage (IV) cases remained the same for all cancers, as no statistically significant differences were noted. However, there was a reduction in stage II/III lung cancer diagnoses in the pandemic period compared to the pre-pandemic period, likely due to catching up on diagnostic activities following the initial wave of the pandemic [41].

### 3.5. Time Trends of the Impact of the COVID-19 Pandemic on Lung Cancer Diagnosis

Several studies reported that the most significant disruptions in lung cancer diagnosis occurred from March to May 2020, primarily due to fear of COVID-19, lockdowns, and social distancing, which led to missed medical appointments. Studies showed 24% of Americans and 15% of British people skipped in-person care during the pandemic. Additionally, healthcare resources were redirected to COVID-19 care, reducing screenings [65]. In Baltimore, USA, lung cancer diagnoses dropped during the first six months of the pandemic but rebounded by mid-2020 [61]. Similar patterns were observed in Italy [40], Japan (Supplementary Material Figure S3) [57], Wales [20], New Jersey (USA) [25], Texas (USA) [84], and Korea [50], with declines in early 2020 followed by partial recoveries, but with further decreases towards the end of 2020 and into 2021 due to lockdowns. Some regions, like Madrid in Spain, saw a rebound in diagnoses, while others continued facing challenges due to ongoing COVID-19 management [85].

### 3.6. Disparities and the Influence of Patient Characteristics on the Impact of the COVID-19 Pandemic on Lung Cancer Diagnosis

We also evaluated the impact of patient characteristics on lung cancer diagnosis during the pandemic. Concerning age, two studies reported that individuals aged 65 and older were most affected by delayed lung cancer diagnoses [18,46]. With regard to gender, in Ukraine and Northern Italy, a higher decline in lung cancer cases was observed in men compared to women (Supplementary Material Figure S4) [17,40]. In Lombardy (Italy), however, the decline was more significant in women under 50 years old (Supplementary Material Figure S4) [19]. Socioeconomic status also had an impact on the way the COVID-19 pandemic affected lung cancer diagnosis. In Hungary [18], during the pandemic, lower lung cancer incidence was observed in areas with below-average median incomes, while in New Jersey (USA) the opposite trend was seen [25].

## 4. Discussion

This study analyzed the impact of the COVID-19 pandemic on lung cancer diagnosis through a systematic review, ultimately basing its conclusions on 78 relevant articles. The COVID-19 pandemic caused various disruptions in healthcare systems, including a reduction in lung cancer incidence, diagnostic services, and screening. Since the disease stage is a crucial prognostic factor, such disruptions can seriously affect the outcome of the disease. Most studies showed a more advanced stage of diagnosis during the pandemic compared to pre-pandemic years for the countries reported (Figure 5 and Supplementary Material Figure S2).

### 4.1. Geographic Variation in the Impact of the COVID-19 Pandemic on Lung Cancer Diagnosis

Taiwan and South Africa showed the highest decline in lung cancer incidence, followed by Spain, the UK, and the USA, compared to other European countries, Canada, and Japan (Figure 2). The observed decrease in lung cancer incidence during the pandemic likely reflects reduced screening and diagnostic activities rather than a true decline in disease incidence. In support of this, lockdowns, social distancing measures, and the redirection of healthcare resources towards COVID-19 management significantly limited access to routine medical care [1,21,66]. Additionally, the decline in lung cancer incidence during the pandemic may be attributed to individuals avoiding medical consultations due to concerns about potential exposure to COVID-19. This trend highlights the critical role organized screening programs play in detecting lung cancer, as disruptions to these programs during the pandemic had a pronounced impact on disease incidence. A systematic review on the impact of the COVID-19 pandemic on cancer revealed declines in the number of cancer screening participation (39.0%), diagnoses (23.0%), diagnostic procedures (24.0%) and treatment (28.0%) during the pandemic compared to pre-pandemic years, supporting the observations discussed above for lung cancer [86]. Finally, the geographic variation in the extent of the decline of lung cancer incidence may be explained by differential usage of CT scans for lung health assessment after COVID-19 infection in different healthcare systems. More specifically, the lower decline in lung cancer incidence in countries like Spain, UK and USA compared to Taiwan and South Africa, may be due to more incidental diagnoses of lung cancer during lung health assessment after COVID-19 infection in these countries, which reduces the overall effect of lung cancer screening disruptions [65,87].

It is essential to note that the availability and implementation of lung cancer screening programs vary widely across countries, contributing to differences in the magnitude of diagnostic disruptions. For example, the USA, which has had a comprehensive lung cancer screening program using LDCT since 2016, experienced significant disruptions in screenings during the pandemic [8]. Furthermore, in the USA there is counseling and shared decision making for LDCT, which adds another roadblock to lung cancer screening during health crises when person-to-person contact is avoided. In contrast, countries without established nationwide programs or those with pilot projects, such as Slovakia, Hungary, and Germany, may have seen relatively smaller declines in lung cancer diagnoses due to the lower baseline participation in screenings. Similarly, the UK offers targeted screening through the NHS and Lung Health Checks in select regions, while countries like Japan, Korea, and China have ongoing screening programs that vary in coverage and implementation (Supplementary Material Figure S5) [8]. According to the results of this study, countries with well-organized programs experienced greater declines in new cases due to screening disruptions, while others with less robust screening systems had smaller declines. The pandemic underscored the importance of maintaining cancer screening even during health crises to prevent delayed diagnoses and increased late-stage cases. These disparities underscore the importance of establishing and maintaining robust screening programs to mitigate the impact of unforeseen challenges like a global pandemic. Addressing barriers such as radiation exposure concerns, accuracy of screening tests, and financial constraints is vital to ensuring that lung cancer screening programs can operate effectively and reach at-risk populations, even during healthcare crises [8].

Furthermore, addressing staffing of the healthcare systems during crises is imperative. More specifically, efforts to maintain a high number of staff during crises would be essential to be able to continue delivering healthcare services. This could be achieved by redirecting medical students and residents to assist in understaffed departments. The capacity of the healthcare systems should also be addressed by expanding physical space and strengthening IT infrastructure so that remote medical consultations can replace hospital visits when possible. Finally, following the COVID-19 pandemic, governments have the opportunity to reflect on healthcare system failures during the pandemic and plan readiness exercises to improve healthcare system responses in future crises [88]. Israel already has a good response system in place during crises, mainly having addressed many of the aforementioned points, and to that end, minimal disruptions in preventive healthcare were observed during the COVID-19 pandemic [89,90].

### 4.2. Increased Lung Cancer Incidence and Incidental Findings

Several studies observed that there was an increase instead of a decrease in lung cancer cases during the COVID-19 pandemic. It appears that the increase in emergency presentations due to the virus led to incidental diagnoses [18,21,65]. Arak et al., in Turkey, reported that incidental lung cancer diagnosis was higher during the pandemic compared to pre-pandemic (23% vs. 9%, *p* = 0.001), and most findings were stage I lung cancer. The time from radiological to histological diagnosis and from histological diagnosis to surgery was similar between groups [87]. However, the time from symptom onset to histological diagnosis was significantly shorter during the pandemic compared to pre-pandemic (median 2 [1,2] vs. 2 [1,2,3] months, *p* = 0.005). Incidental NSCLC diagnoses increased (32 vs. 15, *p* = 0.001), likely due to greater use of thoracic CT imaging. While SCLC was the most common subtype, 72% of incidental cases had adenocarcinoma, possibly because it develops in the lung periphery and remains asymptomatic longer [87]. In addition, a study in Spain by Martinez noted that patients might mistake respiratory symptoms such as persistent cough for COVID-19, prompting them to seek medical advice more quickly than in previous years [83]. This might correlate with the increase in HRCT scans in the ED from 2019 to 2020 in Poland [65]. In 2020, HRCT scans of the chest were performed in 2447 cases (10.1% of ED patients), compared to 780 cases (2.7%) in 2019 (*p* < 0.0001). A total of 217 ED patients were hospitalized with chest tumors in 2019, compared to 181 in 2020. Newly diagnosed chest tumors accounted for 17 cases (0.06% of ED patients) in 2019 and 27 cases (0.11%) in 2020 (*p* = 0.049). Among patients undergoing chest CT, tumors were identified in 2.2% in 2019 and 1.1% in 2020 (*p* = 0.03). The proportion of patients with advanced-stage disease was higher in 2020 (11.8% vs. 29.6%), though, as already mentioned, the difference was not statistically significant [65]. Furthermore, a study by Poghosyan et al., which analyzed the U.S. national rates of lung cancer screening among eligible individuals, showed slight fluctuations over the four years studied between 2019 and 2022 (16.3% in 2019, 19.4% in 2020, 18.3% in 2021, and 18.1% in 2022). Individuals with a history of lung disease or other cancers (excluding lung cancer) were consistently more likely to undergo LCS in all four years [64].

### 4.3. Impact of the COVID-19 Pandemic on the Stage of Lung Cancer at Diagnosis

The increase in advanced-stage diagnoses and concurrent decrease in early-stage detections in countries such as the UK, Italy, Korea, Northern Ireland, Germany, Poland, and Macedonia can be linked to delayed screenings and reduced outpatient visits [21,40,44,50,73,74,75,76]. This increase in advanced-stage disease is expected to have a big impact on public health as it will consequently result in poorer patient outcomes, reduced quality of life, and increased mortality. To this end, one study in the United States has shown slight increases in mortality coinciding with the COVID-19 pandemic [91].

Late-stage lung cancer often presents with symptoms that prompt medical attention, while early-stage cases are typically asymptomatic and reliant on proactive detection through screenings. Conversely, the decrease in advanced-stage cases and increase in early-stage diagnoses reported in Turkey, Denmark, and Brazil may reflect localized adaptations, such as the resumption of screening programs or effective outreach efforts in these regions [46,79,80]. More specifically, these differences between countries may reflect in screening practices and the effectiveness of healthcare systems. A decrease in advanced stages could reflect more effective identification of high-risk individuals and referral of these individuals for screening. For example, in Denmark, the organization of lung cancer diagnosis and treatment as a standardized cancer patient pathway (CPP) with well-defined criteria, along with well-defined procedures and time frames, likely leads to screening of high-risk patients and more effective diagnosis of lung cancer even during the pandemic [79].

### 4.4. Time Trends of the Impact of the COVID-19 Pandemic on Lung Cancer Diagnosis

The most significant disruptions in lung cancer diagnosis occurred from March to May 2020, which coincided with the dates during which most countries had lockdowns [92]. Furthermore, these months of the pandemic were focused on dealing with boosting surveillance systems, re-directing resources to deal with the increasing number of COVID-19 hospitalizations and consequent straining of the hospital systems, which could not cope with preventive and diagnostic services for chronic diseases, including lung cancer. In support of this, a national survey of hospital leaders reported that quality and/or outcomes worsened for outpatient non-COVID-19 patients during the peak of COVID-19 cases [93].

### 4.5. Disparities in Lung Cancer Diagnosis During the COVID-19 Pandemic

Age disparities in lung cancer diagnoses were exacerbated by the pandemic. The disproportionate impact on older individuals (>65 years old) may reflect their heightened vulnerability to delayed diagnoses due to fear of COVID-19 exposure [18,46]. This trend is particularly concerning given that lung cancer is often asymptomatic in its early stages, leading to later-stage diagnoses when symptoms become apparent. Interestingly, a survey in Korea showed that 30% of survey participants reported being more worried by COVID-19 than lung cancer, attesting to the fact that fear of contracting COVID-19 was a deterrent for lung cancer screening [94]. Furthermore, a claims data analysis in Germany regarding general screenings revealed that utilization of individual screenings decreased significantly among the elderly during the COVID-19 pandemic compared to pre-pandemic years, further supporting disparities in screening among this age group during the pandemic [95]. The pandemic may have further delayed screenings and medical consultations, contributing to more advanced-stage diagnoses among older adults. Furthermore, there was an observed higher participation in screenings among younger patients, in California, further supporting age-related disparities in screening rates during the pandemic [51].

Furthermore, gender disparities were observed during the pandemic. More specifically, gender-specific trends, such as decline in cases among men in Ukraine and Northern Italy, may indicate differences between men and women with respect to the health-seeking behaviors or access to screenings [17,40]. Interestingly, the European Health Interview Survey during 2019–2020 revealed that women had significantly higher odds of using outpatient and preventive services, including general practitioners, specialists, dental care, physiotherapy, psychotherapy, and home care, which may explain the aforementioned gender disparities [96]. In this context, there is a need for better health promotion and screening education for men. In contrast, some other studies showed an opposite trend in lung cancer diagnosis, with females showing larger declines. More specifically, a study in Lombardy revealed a larger decline (30% decline) in lung cancer diagnoses among younger women (<50 years old) compared to men (12% decline) of the same age group. However, this trend was reversed for women (16% decline) and men (20%) 50–74 years old. This may be due to the fact that 50–74 year-old individuals are considered to be higher risk for lung cancer and hence may be recommended for screening, with women being more responsive to health provider directions [19]. The aforementioned study potentially suggests that women may have been more attentive to their health during the COVID-19 pandemic, and lack of engagement with the healthcare system was likely due to shortages in the healthcare system and reduction in the capacity for screening. This is supported by a claims data analysis in Germany regarding general screenings, which revealed that utilization of individual screenings decreased significantly among women during the COVID-19 pandemic compared to pre-pandemic years due to interruptions in mammography screening [95]. A study from Portugal found that women avoided healthcare more than men during the same period, however, it should be noted that the study was disproportionate regarding sex, with 74.1% of participants being female, and it did not report the age distribution by sex, thereby making it difficult to reach a solid conclusion regarding gender disparities [97].

Socioeconomic and demographic disparities may also contribute to differences in lung cancer diagnosis during the COVID-19 pandemic. In Hungary, a greater decline in newly diagnosed lung cancer cases was observed in low-income districts, potentially reflecting pre-existing barriers to healthcare access that were further exacerbated during the pandemic [18]. The pandemic had a bigger burden on socioeconomically disadvantaged groups, exacerbating social vulnerabilities and access to healthcare. A study concluded that socially vulnerable communities were less resilient in their capacities to plan for and recover from disruptive events such as the COVID-19 pandemic [97,98]. To this end, in the United States, disparities in LCS uptake across socioeconomic and demographic groups were observed during the pandemic, further re-enforcing the exacerbating effect of the pandemic on the association between socioeconomic and demographic factors and screening behavior. Specifically, Hispanic individuals had lower odds of undergoing LCS compared to Caucasian individuals, and rural residents were less likely to utilize LCS compared to their urban counterparts [64]. These findings underline the need for greater focus on targeted interventions to address inequalities in healthcare access, particularly in underserved communities, which have been exacerbated by the pandemic. This would call for better policies that address social vulnerabilities and provide support to socioeconomically disadvantaged groups to improve healthcare access, especially during times of crises.

### 4.6. The Impact of the Pandemic on Cancers Other than Lung Cancer

Interestingly, lung cancer diagnosis was less affected by the COVID-19 pandemic than other cancers [18,20,28]. A study in Ontario showed weekly declines in diagnoses of cervical (68%), colorectal (33.1%), breast (30.5%), and lung cancer (13.5%) [25]. Additionally, a study in Lombardy reported that lung cancer diagnoses decreased less than other cancers, possibly due to COVID-19 symptoms resembling lung cancer and patients undergoing CT scans to assess lung health due to COVID-19 infection [19]. A variety of reasons could account for this differential impact. One reason, as already mentioned, is the incidental diagnosis of lung cancer due to CT scans conducted for the assessment of COVID-19 patient health. Another reason could be that lung cancer screening is done in high-risk populations, whereas other types of screening are population-level screenings in symptom-free individuals. The population-level screenings were put on hold during the peak of the pandemic, while attempts to deliver services selectively to high-risk individuals were being made for cancers, such as lung cancer, with no population-level screening programs.

### 4.7. Limitations

This study had several limitations. One of the limitations is that some of the articles identified, which may potentially report valuable data, did not provide access to the full article. Moreover, thirteen of the articles identified lacked statistical analysis (*p*-values), therefore they were not included in the analysis. Although the study includes research from various countries, there is no balanced geographical representation. Studies from Western/first-world countries were predominantly presented, making it difficult to generalize the conclusions. This might lead to a biased perspective if some regions are disproportionately represented while others are inadequately covered. Furthermore, there is considerable variability in the sample sizes of the analyzed studies, which should be considered when assessing this data. Additionally, this study has the risk of publication bias. Even though we identified several studies with non-significant findings, there may have been more studies that found no difference and did not publish their results. Furthermore, although all relevant studies were included and discussed in the review, some of them could not be incorporated into the graphical analysis due to differences in data reporting formats or comparison approaches (for example, comparing between specific months rather than broader time periods such as pre-, during or post-pandemic), limiting direct comparability across some studies.

## 5. Conclusions

The COVID-19 pandemic significantly disrupted lung cancer care, affecting diagnosis, screening, and stage at diagnosis. Restrictions led to lower screening rates and increased diagnoses of advanced-stage lung cancer, particularly in areas with higher virus prevalence, which prompted the need for CT scans to assess lung health. Furthermore, expert panels recommended deferring enrollment in lung cancer screening, pending considerations of multiple local, regional, and patient-related factors [15]. Therefore, the pandemic emphasized the need for resilient healthcare systems that can maintain essential services during crises, without disparities in healthcare access, particularly for older and lower socioeconomic status groups. To mitigate the long-term effects of global crises, such as the COVID-19 pandemic, governments and healthcare authorities should consider implementing strategies and protocols that will enable them to offer undisrupted health care services in similar situations. For instance, increasing public awareness regarding lung cancer and educational initiatives through various media platforms or events are essential and can make a difference if coupled with strategic planning and appropriate allocation of resources for systemic preparedness. These initiatives should highlight the risks of delaying referral to diagnostic centers, the benefits of timely action, the importance of participating in screening programs even at times of crises, and provide mechanisms that enable individuals to obtain the healthcare services needed. Strengthening telemedicine services can reduce face-to-face visits as the pandemic strains hospital capacities and assists patients lacking access to diagnostic centers. In addition, as science is progressing and liquid biopsies are gaining ground for the diagnosis of many cancers, including lung cancer [72], the development of highly sensitive and specific blood-based tests should become a priority since their availability will enable continuous lung cancer screening, even at times of crises. Such an approach would not only enhance early detection but it would also reduce the strain on healthcare facilities during critical periods. Efforts must also be made to address ways to mitigate the disparities in individuals’ access to healthcare services, which emerged during the pandemic, through targeted public health interventions and policy changes. Equally important is the execution of more research regarding the impact of the COVID-19 pandemic and other crises on healthcare systems during and post-crises. This will enable healthcare systems to respond better in future crises. Lastly, as screening programs are not available in all countries, governments should invest in providing organized, free or low-cost screening programs to all high-risk individuals. Early diagnosis is key for poor-prognosis diseases, such as lung cancer, and would have a significant social and economic impact by reducing the burden of disease.

## Data Availability

The data can be made available upon request.

## Supplementary Materials

The following supporting information can be downloaded at: https://www.mdpi.com/article/10.3390/cancers18142238/s1, Figure S1: Percentage of cytology for lung cancer in Northern Italy and HRCT scans in Poland in 2019 and 2020 [40,65]; Figure S2: Percentage of lung cancer diagnoses by stage in 2019 and 2020, Milan, Japan, Leicester (UK), and Michigan (USA). The figure shows an overall increase in advanced stage and a decrease in the early stage of lung cancer at diagnosis in the pandemic years compared to pre-pandemic years [44,47,66,78]. The study from Canada [77] is not included in the figure because the study’s data were not directly comparable to the others; Figure S3: Percentage of decline in lung cancer Incidence by month in Japan [58]; Figure S4: Percentage of change in lung cancer Incidence between males and females in Ukraine, Lombardy (Italy), and Northern Italy in 2020 [17,19,40]; Figure S5: Lung Cancer Screening programs around the world [8]. Created in BioRender, Yiallouris, A. (2026) https://BioRender.com/8elvy17 (accessed on 12 June 2026).

## Abbreviations

The following abbreviations are used in this manuscript:

SARS-CoV-2	Severe Acute Respiratory Syndrome Coronavirus 2
COVID-19	Coronavirus Disease
WHO	World Health Organization
PHEIC	Public Health Emergency of International Concern
US/USA	United States of America
UK	United Kingdom
EGFR	Epidermal Growth Factor Receptor
KRAS	Kirsten Rat Sarcoma virus
ALK	Anaplastic Lymphoma Kinase
NSCLC	Non–Small Cell Lung Cancer
SCLC	Small Cell Lung Cancer
CBC	Complete Blood Count
CT	Computed Tomography
LDCT	Low–Dose Computed Tomography
USPSTF	US Preventative Services Task Force
TNM	Tumor–Node–Metastasis
ASCO	American Society of Clinical Oncology
PICOS	Population, Intervention, Comparison, Outcomes and Study
STROBE	Strengthening The Reporting of Observational studies in Epidemiology
CIs	Confidence Intervals
NCRU	National Cancer Registry of Ukraine
LCS	Lung Cancer Screening
Lung-RADS	Lung Imaging Reporting and Data System
RHCs	Rural Health Clinics
HRCT	High Resolution Computed Tomography
ED	Emergency Department
M1c	Multiple Extrathoracic Sites
CP	Control Period
IP	Investigation Period
ECOG PS	Eastern Cooperative Oncology Group Performance Status
SET	Single-Encounter Telemedicine
SIP	Single–visit In Person
MTV	Monthly Screening Test Volume
FOBT	Fecal Occult Blood Test
