# Supplementary material for: Impact of the COVID-19 Pandemic on Lung Cancer Screening and Diagnosis: A Systematic Review
Source: Cancers (Basel). 2026 Jul 13;18(14):2238. doi: 10.3390/cancers18142238 (PMC13406146; doi:10.3390/cancers18142238)
Supplement: Supplementary file 1 [file cancers-18-02238-s001.zip › cancers-4359212-supplementary.pdf]

Supplementary Materials

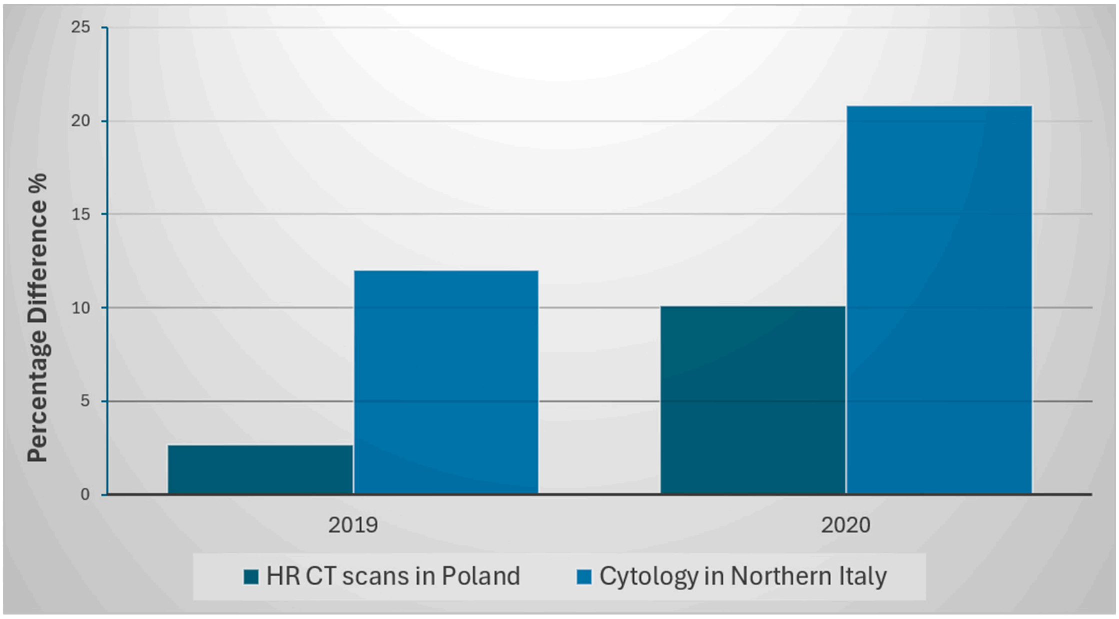

Figure S1. Percentage of cytology for lung cancer in Northern Italy and HRCT scans in Poland in 2019 and 2020 [40,65].

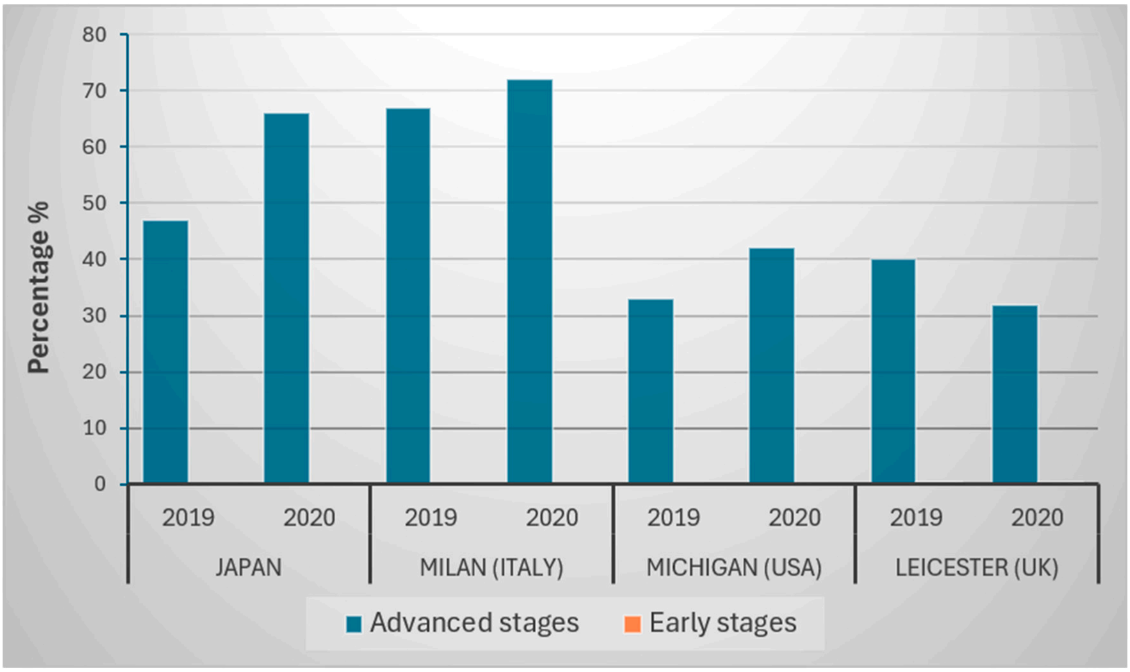

Figure S2. Percentage of lung cancer diagnoses by stage in 2019 and 2020, Milan, Japan, Leicester (UK), and Michigan (USA). The figure shows an overall increase in advanced stage and a decrease in the early stage of lung cancer at diagnosis in the pandemic years compared to pre-pandemic years [44,47,66,78]. The study from Canada [77] is not included in the figure because the study's data were not directly comparable to the others.

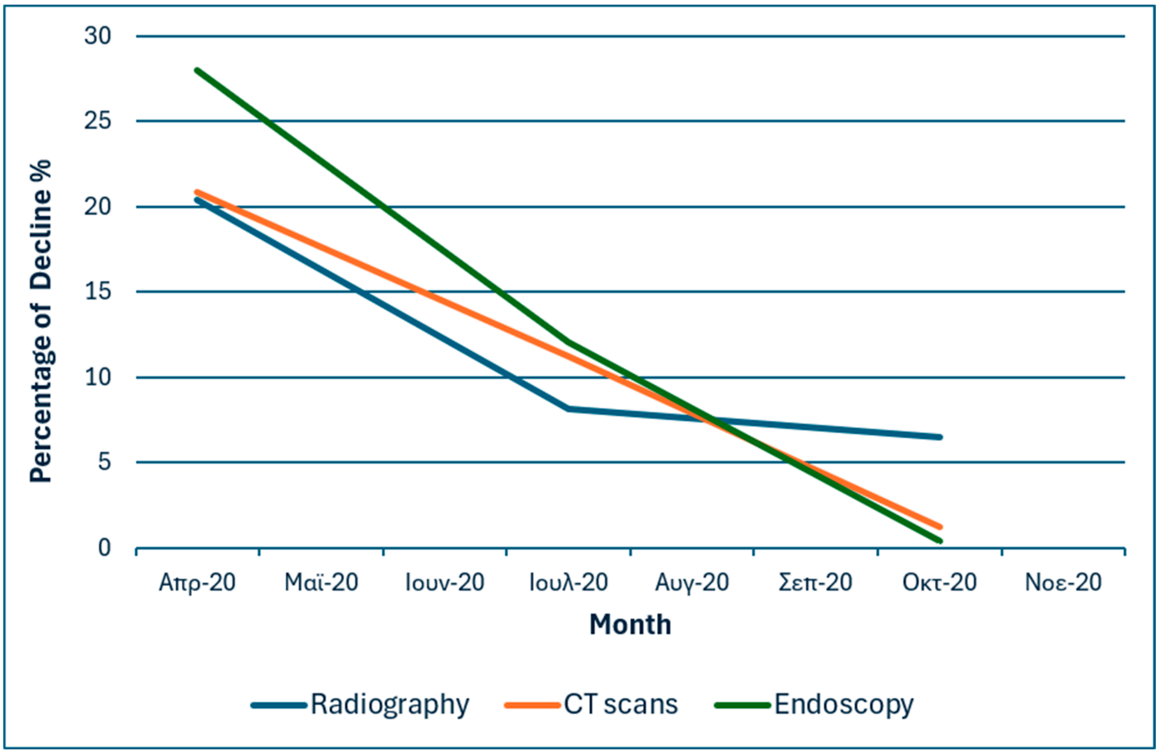

Figure S3. Percentage of decline in lung cancer Incidence by month in Japan [58].

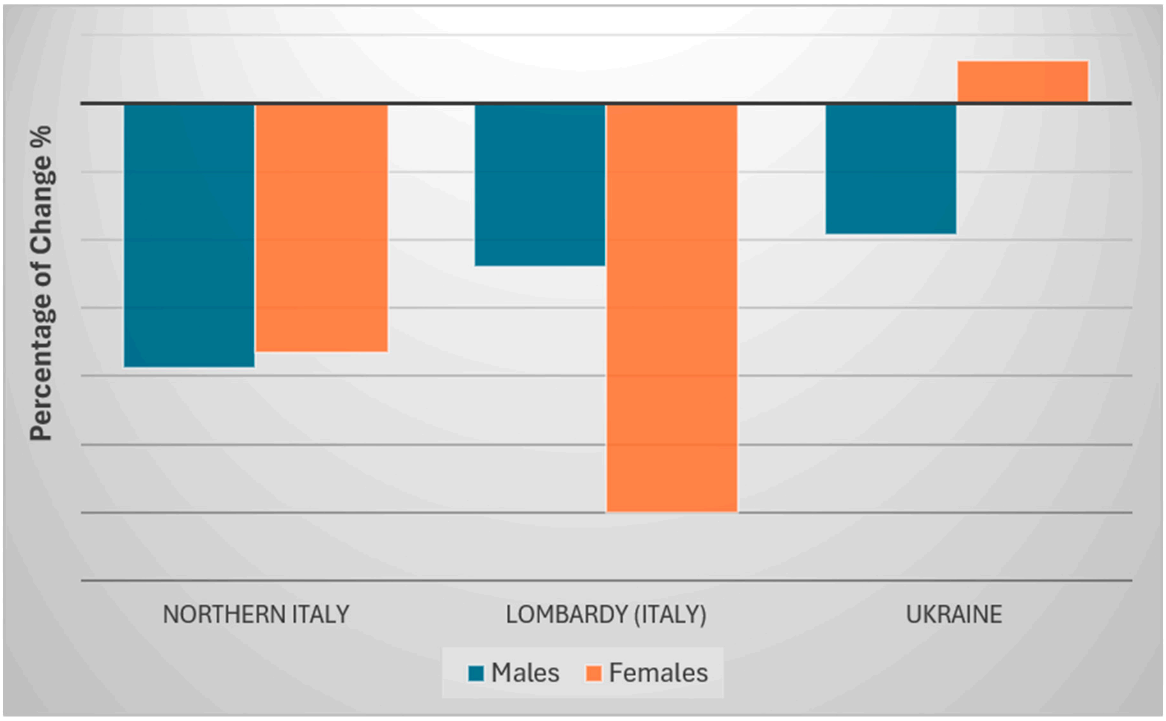

Figure S4. Percentage of change in lung cancer Incidence between males and females in Ukraine, Lombardy (Italy), and Northern Italy in 2020 [17,19,40].

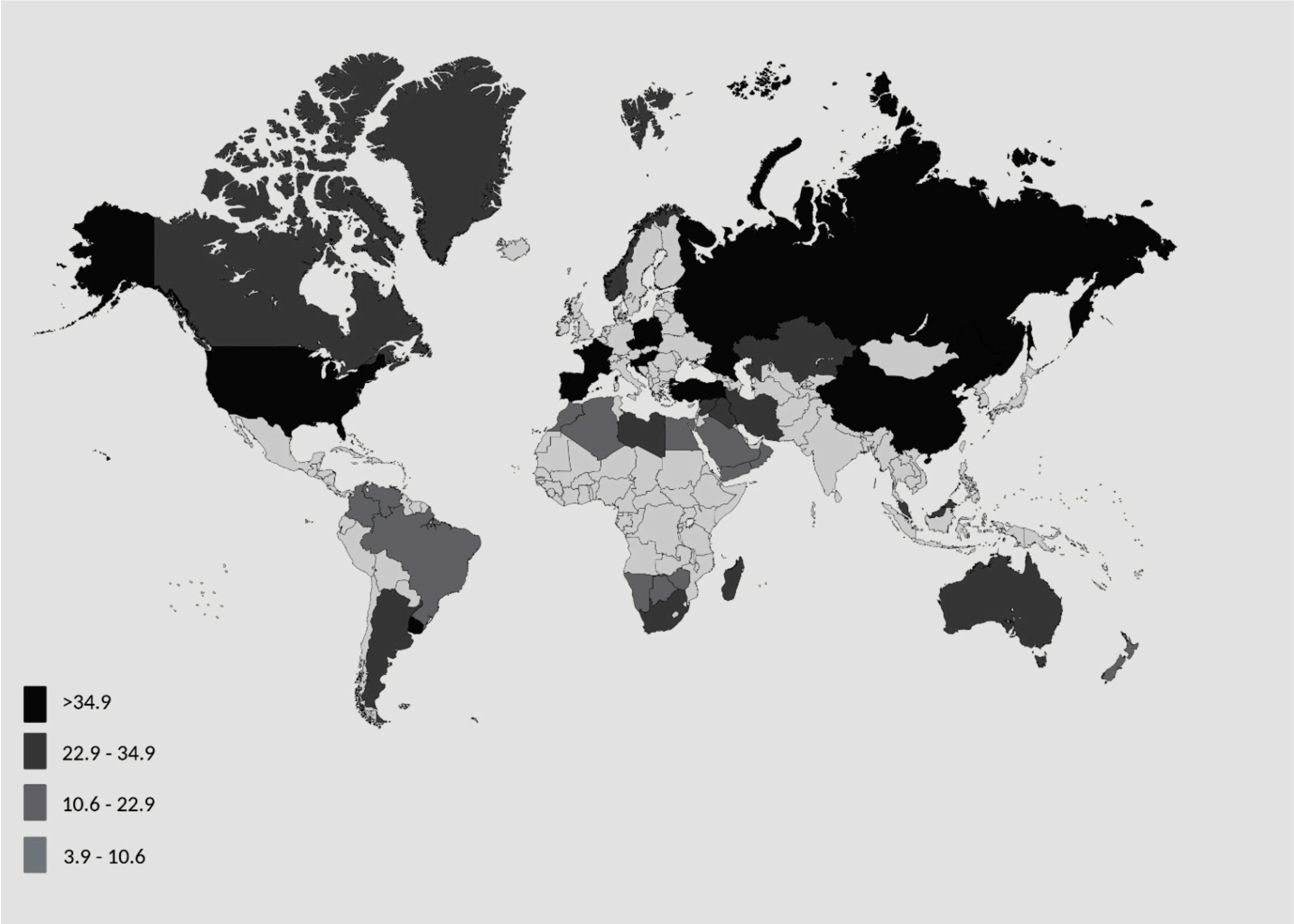

Figure S5. Lung Cancer Screening programs around the world [8]. Created in BioRender, Yiallouris, A. (2026) https://BioRender.com/8elvy17 (accessed on 12 June 2026)
